# Supplementary material for: Macrophages are exploited from an innate wound healing response to facilitate cancer metastasis
Source: Nat Commun. 2018 Jul 27;9:2951. doi: 10.1038/s41467-018-05346-7 (PMC6063977; doi:10.1038/s41467-018-05346-7)
Supplement: Supplementary file 1 — Supplementary Information [file 41467_2018_5346_MOESM1_ESM.pdf]

Macrophages are exploited from an innate wound healing response to facilitate cancer metastasis

Muliaditan et al.

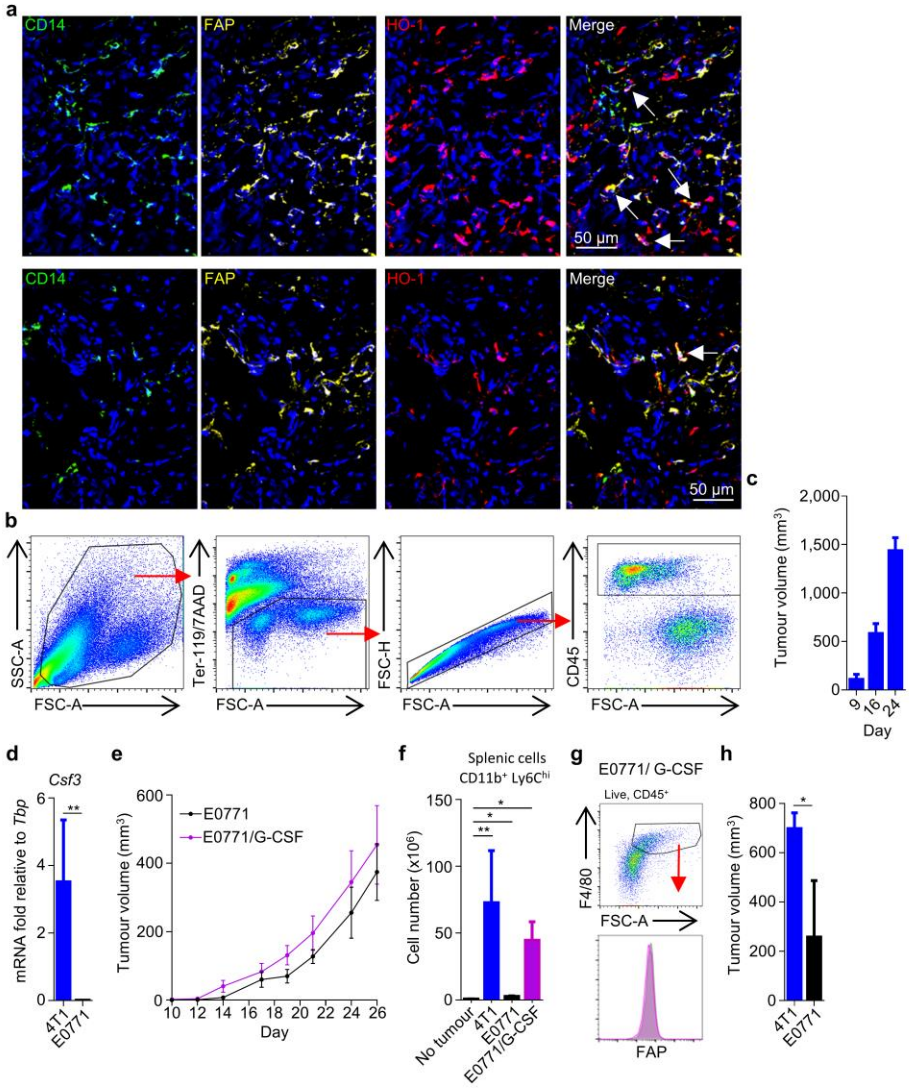

Supplementary Figure 1. FAP⁺ HO-1⁺ TAMs in human and murine breast adenocarcinoma. (**a**) Images of frozen human mammary adenocarcinoma sections stained with DAPI (nuclei; blue) and antibodies against CD14 (green), FAP (yellow) and HO-1 (red). White arrows highlight example cells co-expressing all markers. (**b**) Flow cytometry gating strategy of live $CD45^+$ cells in a representative enzyme-dispersed 4T1 tumour at day 24 post inoculation of tumour cells. (**c**) Tumour volumes at the indicated days post inoculation of 4T1 tumour cells (n = 6). (**d**) Relative *Csf3* mRNA expression in 4T1 (blue, n = 6) and E0771 (black, n = 5) tumours. (**e**) Growth curves of E0771/Thy1.1 (black line, n = 5) and E0771/G-CSF (purple line, n = 6) tumours grown in syngeneic C57Bl/6 mice. (**f**) Absolute quantitation of splenic monocytes ($CD11b^+$ $Ly6C^{hi}$) in tumour free (n = 6), and E0771/Thy1.1 (n = 3), E0771/G-CSF (n = 5) and 4T1 (n = 6) tumour bearing mice at day 24 (E0771/E0771/GCSF) and 26 (4T1) post inoculation of tumour cells, as assessed using flow cytometry analyses. (**g**) Representative FAP staining of live ($7AAD^-$) $CD45^+$ $F4/80^{hi}$ TAMs from enzyme-dispersed E0771/G-CSF tumours as assessed using flow cytometry. Histogram represent positive staining for FAP (purple shaded) against isotype control staining (grey shaded). (**h**) Relative tumour volumes for 4T1 and E0771 tumours concurrently grown in *Rag2*$^{-/-}$ mice at the point of excision (associated with experiment shown in Figure 1k, l) (n = 4). Growth curves are presented as mean ± s.e.m. and bar charts are presented as mean + s.d. * $P < 0.05$,** $P < 0.01$.

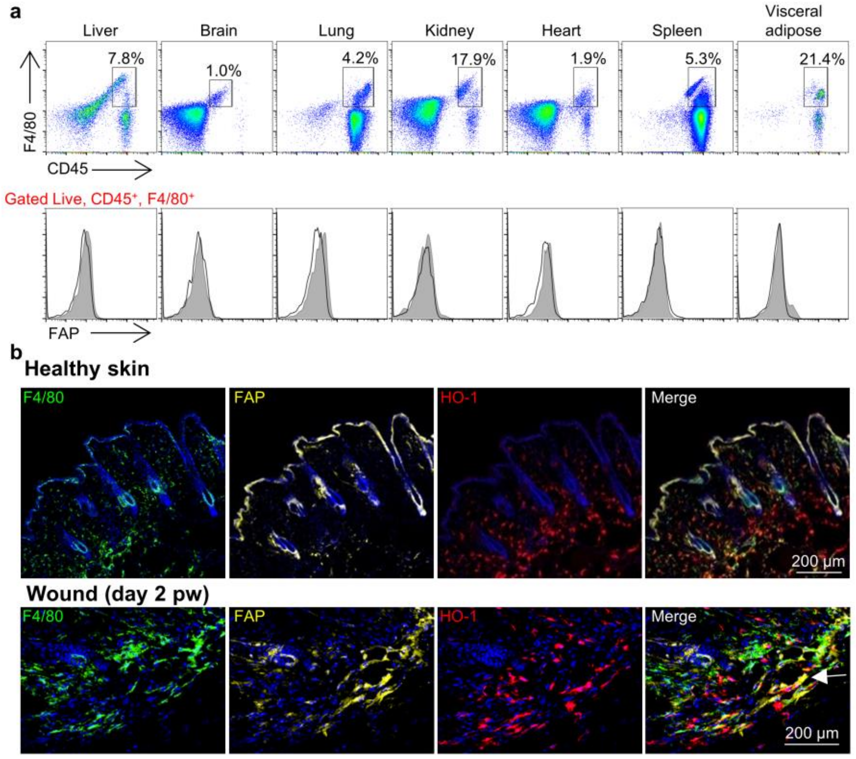

Supplementary Figure 2. Assessment of FAP expression on macrophages in healthy tissues and in the healing wound. (**a**) Representative flow cytometry gating strategy of F4/80$^+$ CD45$^+$ cells (top) and surface FAP expression (open histograms) and isotype staining (shaded histograms) (bottom) in enzyme-dispersed tissues of healthy Balb/c mice. (**b**) Additional images of healthy mouse skin (top panel) and granulation tissue two days post skin wounding using an 8 mm punch biopsy (bottom panel), stained using DAPI (blue; nuclei) and antibodies against F4/80 (green), FAP (yellow) and HO-1 (red).

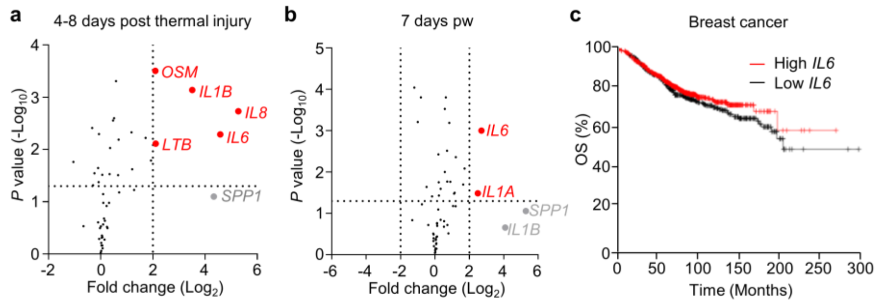

Supplementary Figure 3. Human cytokine gene expression in response to wounding and the prognostic value of *IL6* expression in breast cancer. (**a**) Volcano plot showing changes in cytokine gene expression in human skin 4-8 days after thermal injury[36]. (**b**) Volcano plot showing changes in cytokine gene expression in human skin 7 days following superficial wounding resulting from skin graft donation [37]. Genes marked in red represent cytokines most highly induced in response to wounding ($P \leq 0.05$; fold change ≥ 4). (**c**) Kaplan-Meier survival curves in breast cancer patients showing overall survival (OS) with high (red) and low (black) tumoural expression of *IL6* alone (n = 453 in *IL6*^{lo} group and 949 in *IL6*^{hi} group).

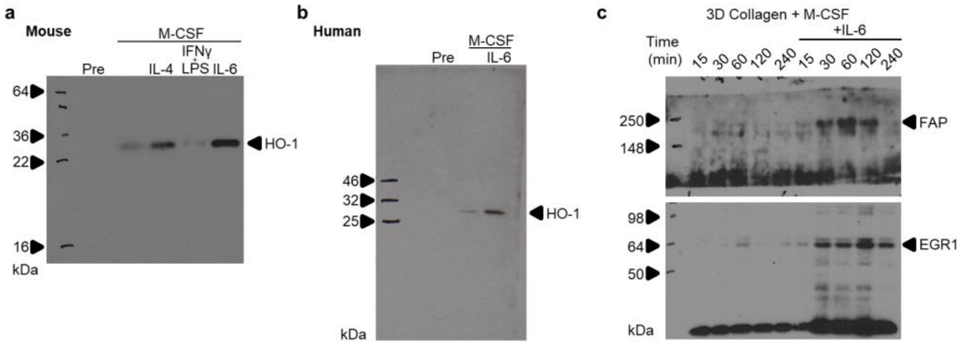

Supplementary Figure 4. Uncropped Western blot images. (**a**) Western blot for HO-1 in BM cells before (Pre) and after 72 h culture in the presence of 50 ng/ml M-CSF with/without 50 ng/ml IL-4, IFNγ + LPS or IL-6. Cropped image shown in Figure 4b top. (**b**) Western blot for HO-1 in human peripheral blood (PB)-derived macrophages before (Pre) and after 72 h culture in the presence of 50 ng/ml M-CSF with/without 50 ng/ml IL-6. Cropped image shown in Figure 4b bottom. (**c**) Western blot analysis for FAP (top), EGR1 (bottom) in BM cells cultured for 72 h with 50 ng/ml M-CSF with/without 50 ng/ml IL-6, and subsequently incubated for indicated times on 3D murine type I collagen. Cropped images shown in Figure 4g.

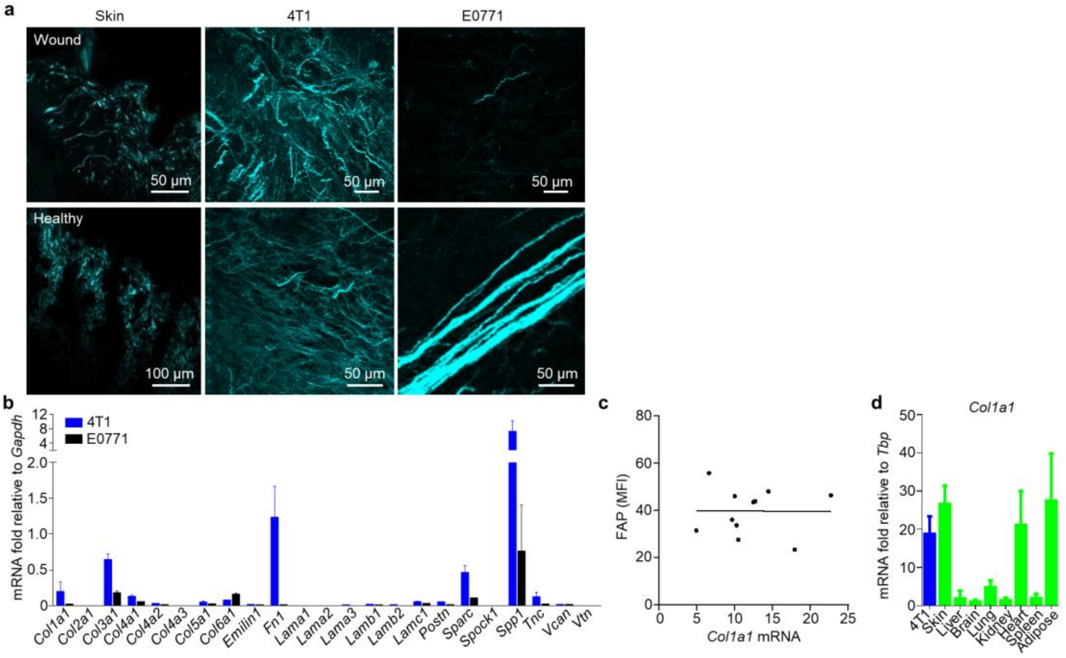

Supplementary Figure 5. Extracellular matrix in the skin and tumour. (**a**) Additional images of SHG imaging of fibrillary collagen in frozen sections of skin 2 days pw and in 4T1 and E0771 tumours (>20 days post inoculation of tumour cells). (**b**) PCR array of expression of genes associated with ECM proteins presented relative to the housekeeping gene *Gapdh*, in 4T1 (blue) and E0771 (black) tumours (n = 2, each pooled from three tumours (>20 days post inoculation)). (**c**) Relative FAP median fluorescence intensity (MFI) of expression by TAMs plotted against that of the respective 4T1 tumoral expression of *Col1a1* mRNA relative to *Tbp* (n = 11). Line of best fit is shown. (**d**) *Col1a1* mRNA expression relative to *Tbp* in 4T1 tumours (n = 5, >20 days post inoculation) and in the respective healthy tissues shown (n = 4). Bar charts show mean + s.d.

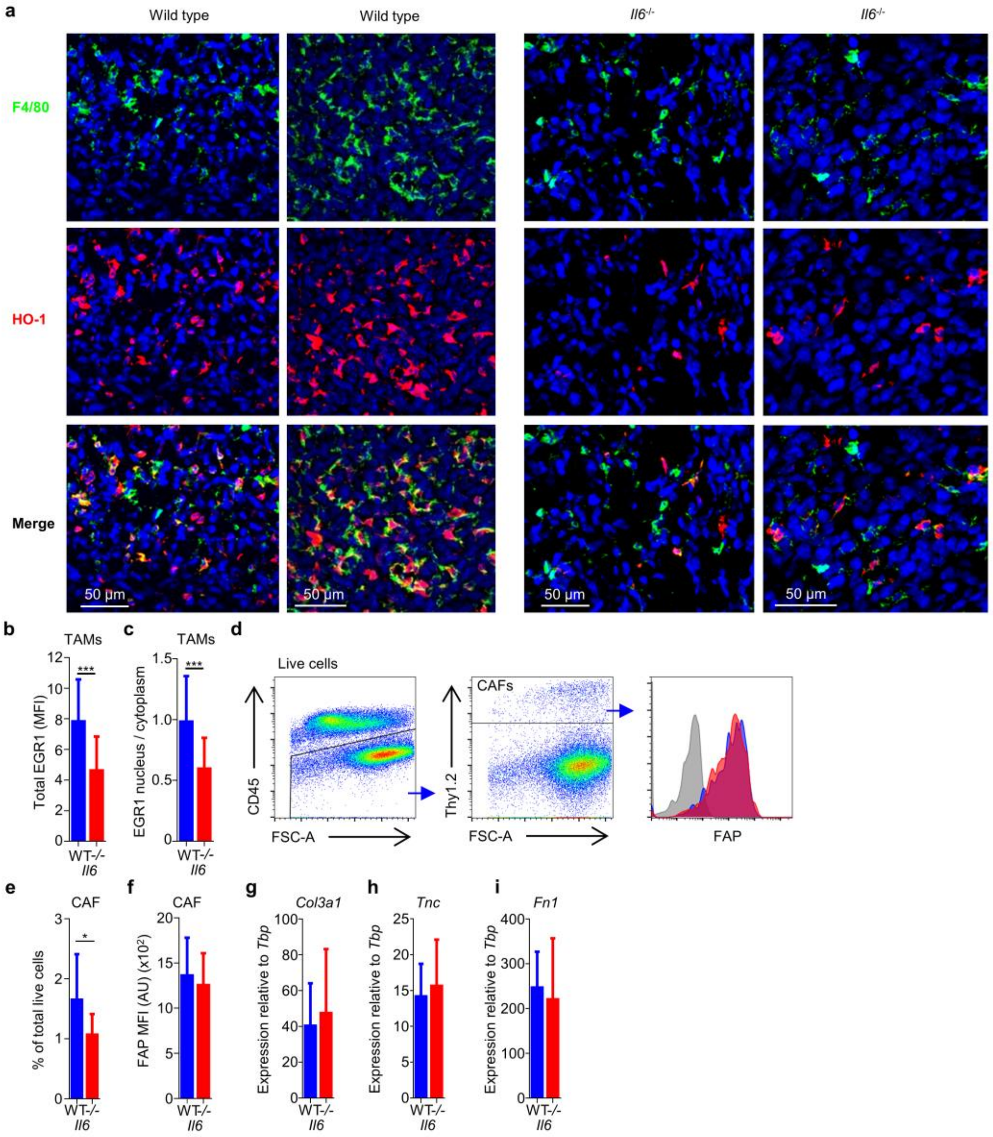

Supplementary Figure 6. Characterisation of WT and *Il6*$^{-/-}$ 4T1 tumours. (**a**) Additional images of frozen sections of 4T1 tumours grown in WT and *Il6*$^{-/-}$ mice at day 23 post inoculation of 4T1 cells, stained with DAPI (blue; nuclei) and antibodies against F4/80 (green) and HO-1 (red). (**b-c**) Quantification of total EGR1 expression based upon median fluorescence intensity (MFI) (b) and the ratio of nuclear and cytoplasmic EGR1 expression (c) in F4/80^{+} TAMs in 4T1 tumours grown in WT or *Il6*$^{-/-}$ mice as determined by confocal microscopy of immunofluorescence-stained frozen sections (n = 20 areas across 3 tumours/group). (**d**) Flow cytometry gating strategy for live (7AAD^{-}) CD45^{-} Thy1.2^{+} CAFs in a representative enzyme-dispersed 4T1 tumour; histogram shows surface FAP staining in WT (blue-shaded) or *Il6*$^{-/-}$ mice (red-shaded) against that of the isotype control (grey shaded). (**e-f**) Abundance of live CD45^{-} Thy1.2^{+} CAFs (e) and their surface FAP expression (f) in enzyme-dispersed 4T1 tumours grown in WT (blue) or *Il6*$^{-/-}$ (red) mice at day 23 post inoculation of tumour cells (n = 11 WT mice and 7 *Il6*$^{-/-}$ mice). (**g-i**) mRNA expression of *Col3a1* (g), *Tnc* (h) and *Fn1* (i) relative to the housekeeping gene *Tbp* in 4T1 tumours grown in WT (blue, n = 8) or *Il6*$^{-/-}$ (red, n = 8) mice at day 23 post inoculation of tumour cells. Bar charts are presented as mean + s.d. * $P \leq 0.05$, *** $P < 0.001$.

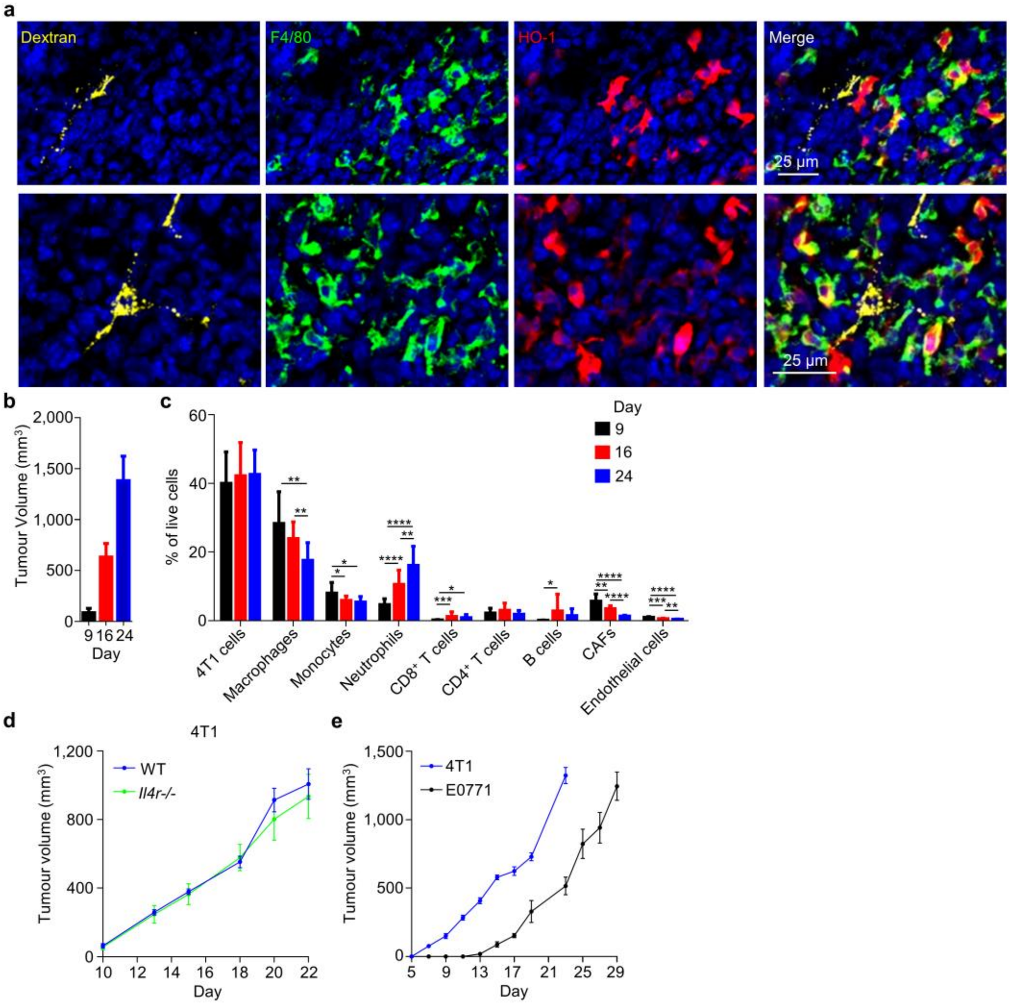

Supplementary Figure 7. Characterisation of 4T1 tumours. (**a**) Additional images of 4T1 tumours stained with DAPI (nuclei; blue) and antibodies against F4/80 (green) and HO-1 (red). Functional vasculature was visualised using i.v. injection of Dextran-FITC (MW 20,000) (yellow) 30 minutes prior to euthanising the mice. (**b-c**) Tumour volumes at the indicated days post inoculation of 4T1 tumour cells (b), abundance of the indicated stromal cells (see Methods for the markers used to differentiate the indicated cells) (c) at the indicated time points (n = 12 day 9, n = 14 day 16, and n = 10 day 24). (**d**) Growth curves of 4T1 tumours grown in syngeneic wild-type mice (WT, blue) or in *Il4r*$^{-/-}$ mice (green) (n = 4). (**e**) Growth curves of 4T1 (blue line) or E0771 (black line) tumours grown in syngeneic WT mice (n = 9). Growth curves are presented as mean ± s.e.m. and bar charts as mean + s.d. * P < 0.05, ** P < 0.01, *** P < 0.001, **** P < 0.0001.

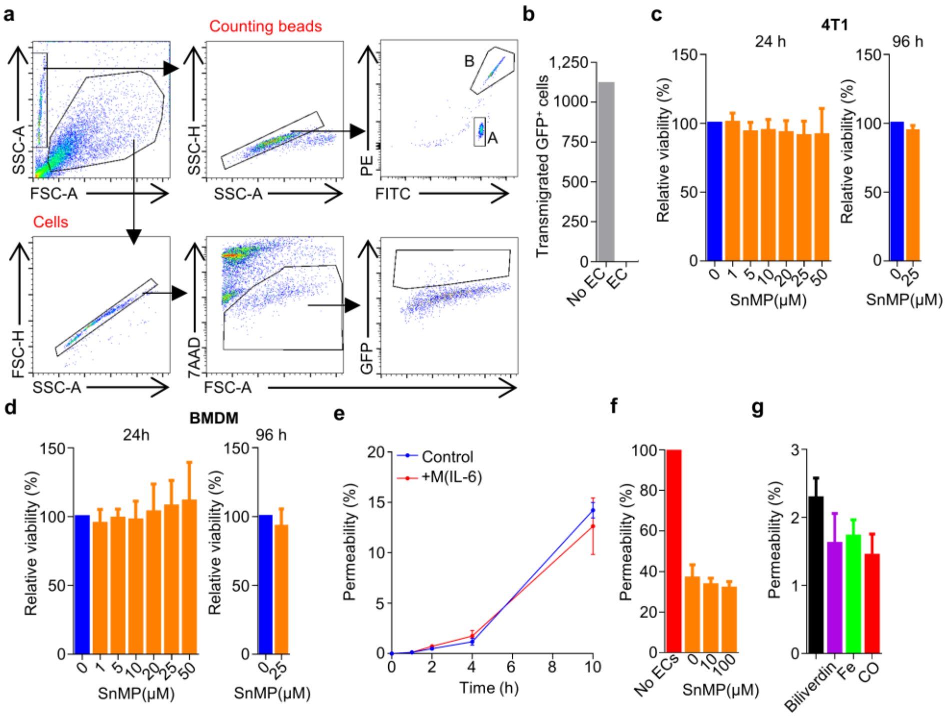

Supplementary Figure 8. The role of HO-1⁺ TAMs in transendothelial migration. (**a**) Flow cytometry gating strategies of 4T1-eGFP cells and counting beads, used to quantify tumour cell transendothelial migration. (**b**) Confirmation of the integrity of the endothelial monolayer in the transendothelial migration assay as assessed by the number of transmigrated 4T1-eGFP cells in 5 h in the absence or presence of an endothelial cell (EC) monolayer (n = 4). (**c-d**) Cell viability of 4T1 cells (c) and BMDM (d) exposed to increasing concentrations of SnMP for 24 h (left panel) and to 25 μM SnMP for 96 h (right panel); viability was assessed using the MTT assay and normalised to vehicle treated cells (n = 6). (**e**) Permeability of an endothelial monolayer to albumin in the presence or absence of M(IL-6) cells (n = 3). (**f**) Permeability of an endothelial monolayer to albumin in the presence of increasing concentrations of SnMP over 24 h (n = 4). (**g**) Permeability of an endothelial monolayer to albumin in the presence or absence of 20 μM Iron (II) chloride, 5 μM biliverdin hydrochloride, or 250 ppm CO (n = 4) over 5 h. Data are presented as mean + s.d.
